# Combining multi-modality data for searching biomarkers in schizophrenia

**DOI:** 10.1371/journal.pone.0191202

**Published:** 2018-02-01

**Authors:** Shuixia Guo, Chu-Chung Huang, Wei Zhao, Albert C. Yang, Ching-Po Lin, Thomas Nichols, Shih-Jen Tsai

**Affiliations:** 1 College of Mathematics and Computer Science, Key Laboratory of High Performance Computing and Stochastic Information Processing (Ministry of Education of China), Hunan Normal University, Changsha, P. R. China; 2 Aging and Health Research Center, National Yang-Ming University, Taipei, Taiwan; 3 Department of Psychiatry, Taipei Veterans General Hospital, Taipei, Taiwan; 4 Division of Psychiatry, School of Medicine, National Yang-Ming University, Taipei, Taiwan; 5 Division of Interdisciplinary Medicine and Biotechnology, Beth Israel Deaconess Medical Center/Harvard Medical School, Boston, United States of America; 6 Institute of Brain Science, National Yang-Ming University, Taipei, Taiwan; 7 Institute of Neuroscience, National Yang-Ming University, Taipei, Taiwan; 8 Department of Statistics, University of Warwick, Coventry, United Kingdom; National University of Defense Technology College of Mechatronic Engineering and Automation, CHINA

## Abstract

Identification of imaging biomarkers for schizophrenia is an important but still challenging problem. Even though considerable efforts have been made over the past decades, quantitative alterations between patients and healthy subjects have not yet provided a diagnostic measure with sufficient high sensitivity and specificity. One of the most important reasons is the lack of consistent findings, which is in part due to single-mode study, which only detects single dimensional information by each modality, and thus misses the most crucial differences between groups. Here, we hypothesize that multimodal integration of functional MRI (fMRI), structural MRI (sMRI), and diffusion tensor imaging (DTI) might yield more power for the diagnosis of schizophrenia. A novel multivariate data fusion method for combining these modalities is introduced without reducing the dimension or using the priors from 161 schizophrenia patients and 168 matched healthy controls. The multi-index feature for each ROI is constructed and summarized with Wilk's lambda by performing multivariate analysis of variance to calculate the significant difference between different groups. Our results show that, among these modalities, fMRI has the most significant featureby calculating the Jaccard similarity coefficient (0.7416) and Kappa index (0.4833). Furthermore, fusion of these modalities provides the most plentiful information and the highest predictive accuracy of 86.52%. This work indicates that multimodal integration can improve the ability of distinguishing differences between groups and might be assisting in further diagnosis of schizophrenia.

## Introduction

Schizophrenia is a major mental disorder characterized by positive and negative symptoms, as well as persistent neuro-cognitive deficits. Considerable efforts have been made over the last decades to identify biological markers for clinical diagnosis and prediction of disease progression. Plenty of studies have described significant differences in schizophrenia (SZ) patients compared to healthy controls (HC)across different imaging modalities. Each modality records specific facets of structure or function MRI and provides biomarkers that may either be specific or common to other modalities. Typically, these modalities are analyzed separately based on their own models and thus the biomarkers are obtained from each single modality separately. For example, functional MRI (fMRI) measures the hemodynamic response related to neural activity in the brain dynamically, from which disease biomarkers related to dorsolateral prefrontal cortex, anterior cingulate cortex, and parietal cortex are generally reported [1–5]. Structure MRI (sMRI) provides information about the tissue type of the brain (grey matter (GM) and white matter (WM)), from which biomarkers related to frontal and temporal cortex, and hippocampus are often pointed out [6–9]. Diffusion tensor imaging (DTI) captures diffusion of water molecules to reflect the directional microstructure within WM and complements the missing orientation information in structural images. By using DTI and related analysis, abnormalities related to superior and prefrontal cortex, temporal cortex, and anterior cingulate cortex in schizophrenia patients are frequently studied [10,11]. Despite the imaging studies have identified quantitative alterations in schizophrenia, these findings have not yet provided a diagnostic measure with sufficient high sensitivity and specificity. Even though part of the actualities is the heterogeneousness of these psychiatric disorders, the lack of consistent findings due to the specificity of individual model also limits the sensitivity and specificity. It thus misses important differences which are only partially detected by each modality [12].

Recently, collection of multimodal brain images from each individual has become common practice, which takes advantage that each modality provides a unique dimension of the brain. This motivates the need for a joint analysis of these multi-modality data, which may uncover previously hidden relationships that can unify disparate findings in brain imaging [12]. Comparison between function and structure provides more informative insights into both altered brain patterns and connectivity, and might be useful for diagnosis of schizophrenia when having both [13–15]. For example, one would detect a change in functional magnetic resonance imaging (fMRI) activation maps that are associated with a change in the brain structure [16,17]. A lower and different function–structure connectivity is often found in patients with schizophrenia compared with healthy controls [18,19], suggesting that combination of two imaging modalities provides more comprehensive descriptions of the altered connectivity.

One existing approach for combining or fusing data in brain imaging includes constraining one modality with another, as DTI being constrained by fMRI or sMRI data [20], or vice versa. While this is a powerful technique, a common limitation is that the potentially unrealistic assumptions which are fundamentally of a different nature than the known modality would be imposed upon the constrained data. Another data fusion approach is to first process each image type and extract features from different modalities. These features are then examined for relationships among the data types at the group level (i.e., variations among individuals or between patients and controls). Such approaches include those based upon singular value decomposition (SVD) and partial least squares (PLS) [21,22] as well as joint independent component analysis(jICA) [12], multimodal canonical correlation analysis (mCCA) [23,24], linked ICA [25] and other adaptive approaches such as parallel ICA [26]. These approaches take advantage of the ‘cross’-information among data types when performing multimodal fusion and provides a natural link among different data types. However, the data matrices are transformed into a smaller set of components in order to reduce the dimension with these approaches, which may result in a loss of important information [27].

Moreover, most current approaches focuses on pair-wise fusion [12, 19, 24, 28–29]. Only very few studies focuses on examining the relationships among multiple data types by using dimension-reduction data fusion approaches, such as mCCA, jICA, etc [16,17]. Given the power of modern MR scanners, more than two imaging modalities are generally available for each participant, multi-way fusion joint multivariate analysis of multiple data types (e.g., resting state fMRI, task-related fMRI, DTI, and sMRI) might improve the ability to classify brain diseases and to detect more informative biomarkers in mental disorders such as schizophrenia.

Hereby, we develop a novel multivariate fusion method to combine the resting-state fMRI (rfMRI), sMRI and DTI without reducing the dimension or using prior information. The multi-index feature for each ROI is first constructed and summarized with Wilk's lambda by performing multivariate analysis of variance to detect significant changes between different groups. Considering the importance of multimodal features, the method we proposed should enable examination of full correspondence across three modalities by achieving reliable inter-modality associations and achieving high predictive power for brain disorders.

## Methods

### Subjects

One hundred and sixty-one patients with chronic schizophrenia (66 males and 95 females) were recruited for this study and they were identified according to the DSM-IV diagnostic criteria by qualified psychiatrists at the Taipei Veterans General Hospital. Exclusion criteria included the presence of DSM-IV Axis I diagnoses of other disorders such as bipolar disorder, history of any substance dependence or history of clinically significant head trauma. Illness durations ranged from several months to 52 years (mean±SD: 17.86±11.1 years). All patients were being treated with a range of antipsychotics. Their average age was 44.99±11.5years and they had a mean education duration of 12.33±3.66 years. Symptom severity was measured using the Positive and Negative Syndrome Scale (PANSS) assessment which was given to all schizophrenic participants either one week before the MRI scan or one week after it. All patients complete their PANSS assessment as listed in Table 1.

**Table 1:** Subject demographics.

	Schizophrenia patients n = 161		Controls n = 168	P value
Age (year)	44.99±11.5	43.17±10.8		0.14	
Education (year)	12.33±3.66	15.8±3.5		<0.001	
Sex (M/F)	66/95	72/96		0.73	
IIlIllness duration(year)	17.86±11.1	n.a.		n.a.	
P PANSS-positive scale	9.6±3.3	n.a.		n.a.	
PANSS-negative scale	9.96±5.6	n.a.		n.a.	
PANSS-general psychopathology scale	20.7±4.8	n.a.		n.a.	
PANSS- Total	40.3±11.2	n.a.		n.a.	
MMSE	26.9 ±3.3	n.a.		n.a.	

Note: Demographic information for the patient and control groups. Mean and standard deviation are provided for continuous variables (e.g., age, education, and PANSS scales). PANSS = Positive and Negative Syndrome Scale.

One hundred sixty eight(72 male and 96 female) healthy control subjects were also recruited. Their average age was 43.17±10.8 years, and their mean education duration was 15.8±3.5 years. All of the controls were assessed in accordance with DSM-IV criteria as being free of schizophrenia and other Axis I disorders. None of them had any neurological diseases or suffered from clinically significant head trauma and none had a history of any substance dependence. The patient and control groups were well matched by gender (χ^2^ = 1.2, p = 0.73) and age (t = 1.48, p = 0.14) although the controls had a slightly longer education duration (t = -8.8, p<0.001). Patient and healthy control demographics are shown in Table 1.

All of the patients were diagnosed according to the Diagnostic and Statistical Manual of Mental Disorders-IV criteria, then administered a diagnostic structured Mini- International Neuropsychiatric Interview (MINI), Mini-Mental State Examination (MMSE) Chinese version. The cognitive functioning of the participants was evaluated using the MMSE for general cognitive status and the Wechsler Digit Span subtest for verbal working memory abilities. All participants exhibited sufficient visual and auditory acuity to undergo cognitive testing. This study was conducted in accordance with the Declaration of Helsinki and approved by the Institutional Review Board of Taipei Veterans General Hospital. Written informed consent was obtained from all participants ensuring adequate understanding of the study. Any participants with the presence of possible dementia or illiteracy were excluded.

## Imaging acquisitions and data preprocessing

### Imaging parameters

All images were acquired using a 3T MR system (Siemens Magnetom Tim Trio, Erlangen, German) at National Yang-Ming University, equipped with a high-resolution 12-channel head array coil. To minimize the head motion during the scan, each subject’s head was immobilized with cushions inside the coil during the scan. A high-resolution anatomical T1-weighted image was acquired with sagittal 3D magnetization-prepared rapid gradient echo (MPRAGE) sequence: repetition time (TR) = 3500 ms, echo time (TE) = 3.5 ms, inversion time = 1100 ms, flip angle = 7°, field of view (FOV) = 256 × 256 mm^2^, 192 slices, slice thickness = 1 mm, voxel size = 1×1×1 mm^3^. The diffusion images gradient encoding schemes include 30 non-collinear directions (according to the minimal energy arrangement of electron distribution (http://www2.research.att.com/~njas/electrons/dim3) with b-value 1000 s/mm^2^, number of excitations: 3) and 3 non-diffusion weighted image volume. With the consideration of total brain coverage, each volume consisted of 70 contiguous axial slice (thickness: 2 mm) acquired using a single shot spin-echo planar imaging (EPI) sequence (TR: 11,000ms, TE: 104ms, NEX: 6, Matrix size: 128×128, voxel size: 2×2×2 mm^3^, matrix size: 128×128). For resting state fMRI: T2-weighted images with BOLD contrast were measured using a gradient echo- planar imaging (EPI) sequence (repetition time, TR: 2,500 ms, echo time, TE: 27 ms, field of view, FoV: 220 mm, flip angle: 77 degree, matrix size: 64×64, and voxel size: 3.44×3.44×3.40 mm^3^), participants were instructed to relax with their eyes closed, without falling asleep during the total 8min scan time. For each run, 200 EPI volume images were acquired along the anterior and posterior commissure (AC–PC) plane. After the resting state experiment, participants were asked whether they fell asleep during the resting state scan session, and the participants were rescanned if they slept during the resting state scan.

### fMRI preprocessing

fMRI data preprocessing was then conducted by SPM8 (University College London,UK; http://www.fil.ion.ucl.ac.uk/spm) and DPARSF(Data Processing Assistant for resting-state fMRI). Briefly, the remaining functional scans were first corrected for within-scan acquisition time differences between slices, and then realigned to the middle volume to correct for interscan head motions. Subsequently, the functional scans were spatially normalized to a standard template (Montreal Neurological Institute) and resampled to 3×3×3mm^3^. After normalization, the Blood Oxygenation Level Dependent(BOLD) signal of each voxel was first detrended to abandon linear trend and then passed through a bandpass filter (0.01–0.08 Hz) to reduce low-frequency drift and high-frequency physiological noise. Finally, nuisance covariates including head motion parameters, global mean signals, white matter signals and cerebrospinal fluid signals were regressed out from the Blood Oxygenation Level Dependent signals.

### sMRI preprocessing

All T1-weighted structural data were pre-processed using the Diffeomorphic Anatomical Registration using Exponentiated Lie algebra (DARTEL) toolbox [30] in SPM8 software (http://www.fil.ion.ucl.ac.uk/spm) running under Matlab (MathWorks, USA). This procedure involves the creation of a study-specific template and the segmentation of each individual image using said template, with the aim of maximizing accuracy and sensitivity [31]. After modulate normalizing, the images were segmented into gray matter, white matter and the cerebrospinal fluid. These segmented images were smoothed using a 12-mm full width at half maximum (FWHM) Gaussian kernel.

### DTI preprocessing

Before image data processing, one author (Chu-Chung Huang, an experienced radiological technician) received all of the MRI scans to confirm that the participants were free of any morphological abnormalities and apparent WM lesions. Eddy current correction and brain tissue extraction of DTI dataset were pre-processed using FSL 5.0.9 (Functional Magnetic Resonance Imaging of the Brain Software Library; http://www.fmrib.ox.ac.uk/fsl). Eddy current correction involved registering the diffusion-weighted images to the non-diffusion weighted image through affine transformations. It was done not only to minimize image distortion derived from eddy currents induced by fast-switching gradient coils, but also to reduce the simple head motion. Subsequently, the Brain Extraction Tool (BET) compiled in FSL was applied to remove the non-brain tissue and background noise from the images. After DTI preprocessing, parameter maps include FA (relative ratio of axial to radial diffusivities), RD (radial diffusivity, (*λ*_2_+*λ*_3_)/2 and MD (mean diffusivity, (*λ*_1_+*λ*_2_+*λ*_3_)/3) were calculated by fitting Baser’s DTI tensor model [32]at each voxel using in-house software. The definitions of *λ*_1_, *λ*_2_, *λ*_3_ are given in Supplemental Materials.

### Construction of multi-index vector for each ROI

After preprocessing, each modality is first reduced to a “feature” for each subject, which tends to be more tractable than working with the large-scale original data and provides a simpler space to link the data [33]. In this paper, an automated anatomical labeling atlas [34]was used to parcellate the brain into 90 regions of interest (ROIs) (45 in each hemisphere) and the “feature” is obtained for each ROI. The names of the ROIs and their corresponding abbreviations are listed in S1 Table.

**Fig 1:**
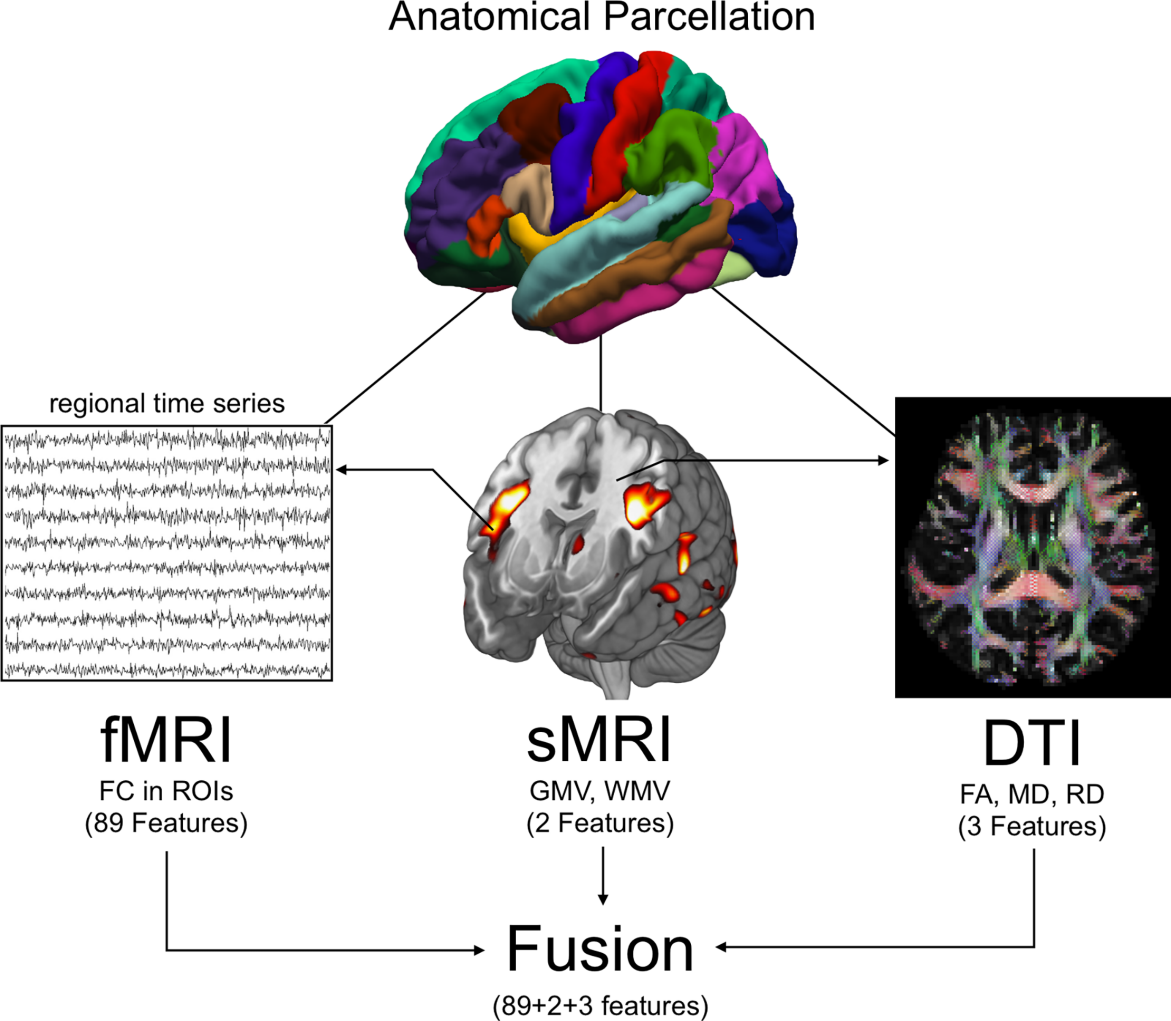
The flowchart of data fusion in this paper.

For fMRI, the time series were firstly extracted in each ROI by averaging the signals of all voxels within that region. Secondly, ordinary Pearson correlation coefficients with all other 89 different ROIs were calculated for each ROI. A Fisher’s r-to-z transformation was utilized to convert each correlation coefficient *r*_*ij*_ into *Z*_*ij*_ to improve the normality. A vector consisting of all these 89 functional connectivity (FC) coefficients (*Z*_*ij*_) was constructed as the functional measure (fMRI) for each ROI. For sMRI, the volume of grey matter (GM) and white matter (WM) was extracted as the structural feature for each ROI. For DTI, the fractional anisotropy (FA), radial diffusivity (RD) and mean diffusivity (MD) were extracted as the DTI features for each ROI. By combining with the functional, structural and DTI features, a vector consisting of 94 variables (89 fMRI features, 2 sMRI features, and 3 DTI features) was constructed as multi-index vector for each ROI. To avoid being overly biased by data scaling, the combined data was normalized by subtracting the mean and dividing by the standard deviation. The flowchart of the fusion data is shown in Fig 1.

### Multivariate analysis of variance

Multivariate analysis of variance (MANOVA) is one of the multivariate approaches. It is a statistical test procedure for comparing multivariate (population) means of several groups. The multivariate approach has been adapted for single-mode fMRI data [35,36], sMRI data [37] and DTI data [38] in earlier studies.

Suppose there are *G* different groups (patient, healthy control, etc) with *n*_*g*_ individuals in the *g*-th group. For each individual k in the *g*-th group, there are m measures to form a measurement vector

$$X_{gk}={\left[x_{gk}^{\left(1\right)},x_{gk}^{\left(2\right)},\ldots ,x_{gk}^{\left(m\right)}\right]}^{T},g=1,2,\ldots ,G;k=1,2,\ldots ,n_{g};n_{1}+n_{2}+\ldots +n_{G}=n$$

where $x_{gk}^{\left(i\right)}$ is the *i-*th measurement of the *k*-th sample on the *g*-th group. We assume that *X*_*gk*_ follows a multivariate normal distribution *X*_*g*_ ∼ *N*_*m*_(*μ*_*g*_, ∑),*g* = 1,2,…, *G*

In order to test the ability of each ROI to distinguish the different groups, we define the following measures: the within-group dispersion matrix *W* = [*w*_*ij*_]_*m*×*m*_, the between-group dispersion matrix *B* = [*b*_*ij*_]_*m*×*m*_ and the total dispersion matrix *T* = [*T*_*ij*_]_*m*×*m*,_ where

$$w_{ij}=\overset{G}{\underset{g=1}{\sum }}\overset{n_{g}}{\underset{k=1}{\sum }}(x_{gk}^{\left(i\right)}-{\bar{x}}_{g}{}^{\left(i\right)})(x_{gk}^{\left(j\right)}-{\bar{x}}_{g}{}^{\left(j\right)})$$

$$b_{ij}=\overset{G}{\underset{g=1}{\sum }}n_{g}({\bar{x}}_{g}^{\left(i\right)}-{\bar{x}}^{\left(i\right)})(x_{g}^{\left(j\right)}-{\bar{x}}^{\left(j\right)})$$

$$t_{ij}=\overset{G}{\underset{g=1}{\sum }}\overset{n_{g}}{\underset{k=1}{\sum }}(x_{gk}^{\left(i\right)}-{\bar{x}}^{\left(i\right)})(x_{gk}^{\left(j\right)}-{\bar{x}}^{\left(j\right)})$$

$${\bar{x}}_{g}^{\left(i\right)}=\frac{1}{n_{g}}\overset{n_{g}}{\underset{k=1}{\sum }}x_{gk}^{\left(i\right)}(g=1,2,\ldots ,G),$$

$${\bar{x}}^{\left(i\right)}=\frac{1}{n}\sum_{g=1}^{G}\sum_{k=1}^{n_{g}}x_{gk}^{\left(i\right)}$$

The ordinary Wilk's Lambda statistic was defined as follows:

$$\lambda =\frac{\left|W\right|}{\left|T\right|}$$

The smaller the value of *λ* is, the bigger the difference of different groups are.

In order to calculate the *p* value of each Wilk’s Lambda statistics, we transform it to a chi-square distribution with the following formulation:

$$\chi^{2}=-(n-1-(G+m)/2\cdot \log(\lambda )$$

Bartlett showed that if *H*_0_: *μ*_1_ = *μ*_2_ = … = *μ*_*G*_ is true and *n* is large, *λ* has approximately a chi-square distribution with *m*×(*G*−1) degrees of freedom[39]

### Support vector machine (SVM) classifier

The SVM is a machine learning approach for a two-class classification problem. Since first proposed by Vapnik as a logistical extension of statistical learning theory, SVM has become widely used in many areas because of their ability to handle very high-dimensional data, and their accuracy in classification and prediction. SVM has become increasingly used in studies of psychiatric and neurological disorders[40],the rationale for which is that it is able to account for the inter-relationship between different within-modality measures for each subject by considering them simultaneously.

SVM conceptually implements the idea that vectors are non-linearly mapped to a very high dimensional feature space. In the feature space, a linear separation surface is created to separate the training data by maximizing the margin between the vectors of the two classes. The training ends with the definition of a decision surface that divides the space into two sub-spaces. Each sub-space corresponds to one class of the training data. Once the training is completed, the test data are mapped to the feature space. A class is then assigned to the test data depending on which sub-space they are mapped to.

In this paper, a SVM toolkit named libsvm written by Chih-Jen Lin from Taiwan university [41](http://www.csie.ntu.edu.tw/~cjlin/libsvm/) is used. Specifically, the whole brain single-modal or multi-modal features are applied to the raw input matrix. Statistically significant features (p-value of two-sample t-test being smaller than a threshold) are selected. Different kernel types (linear, t = 0; polynomial, t = 1; radial basis function, t = 2) and different trade-off parameter C (0.001, 0.01, 0.1, 1, 10, 100, 1000, 10000) is tried to obtain the highest accuracy rate. To measure the test performance and to validate the classifier, we implement 10-fold cross-validation with three levels of nesting for tuning and validating our model. That is, for each iteration, eight folds of the subjects are used for training, one fold is used to optimize the parameters and another fold for testing. The procedure is iterated for 100 times, and the meandiscrimination accuracy, sensitivity and specificity are reported with the best parameter setting. The features are selected in each cross-validation run and the features with high weights during the 100 iterations are the optimized features for each classifier. Choosing the generalization rate as the statistic, permutation tests are employed to estimate the statistical significance of the observed classification accuracy. In permutation testing, the class labels of the training data are randomly permuted prior to training. Cross-validation is then performed on the permuted training set, and the permutation is repeated 100 times. The *p-*value represents the probability of observing a classification prediction rate in a permutation testing no less than the discrimination accuracy. If the *p-*value is smaller than the significant level, we reject the null hypothesis that the classifier could not learn the relationship between the data and the labels reliably and declare that the classifier learns the relationship with a probability of being wrong of at most *p*[42].The flowchart of the pattern recognition is shown in S1 Fig.

### Diversity of individual modalities in classification

As mentioned earlier, a lot of studies have indicated that different modalities contain complementary information for discrimination [43]. In order to quantitatively measure the discrimination similarity and diversity between any two different modalities, we use Jaccard similarity coefficient [44] and Kappa index [45] to achieve these goals.

The Jaccard coefficient measures similarity between finite sample sets, and is defined as the size of the intersection divided by the size of the union of the sample sets. Cohen's kappa measures the agreement between two raters who each classify *N* items into *C* mutually exclusive categories. The equation for kappa is:

$$K=\frac{\mathrm{Pr}(a)-\mathrm{Pr}(e)}{1-\mathrm{Pr}(e)}$$

Where Pr(*a*) is the relative observed agreement among raters, and Pr(e) is the hypothetical probability of chance agreement, using the observed data to calculate the probabilities of each observer randomly staying each category.

### Contribution of different modalities

When scanning the same subject, we can obtain fMRI, sMRI and DTI at the same time. The multi-modal data provides a different perspective on the study of brain function and structure. In order to investigate which kind of modality is more powerful, we intend to study the contribution of each modality to the fusion data. Suppose *X*_*i*_ is the features for subject *i*. Then we use the following logistic regression to model the probability, *p*_*i*_, for the *i*-th subject to be a patient:

$$\log(\frac{p_{i}}{1-p_{i}})=X_{i}^{T}\beta +\varepsilon_{i}$$

In order to assess the contribution of different modalities, we use *R*^2^ indices, defined by Cox and Snell [46] to describe how well the features *X*_*i*_ fit a set of observations (i.e., goodness of fit). We denote $R_{fusion}^{2}$, $R_{fMRI}^{2}$, $R_{sMRI}^{2}$, $R_{DTI}^{2}$ as the *R*^2^ indices of four different models when we use fusion, fMRI, sMRI, and DTI features as independent, respectively. We define the contribution of different modality to fusion data as the ratio of *R*^2^ of each modality to *R*^2^ of fusion data. For example, the ratio $\frac{R_{fMRI}^{2}}{R_{fusion}^{2}}$ is the contribution of fMRI data.

## Results

### Comparison of individual modalities vs fusion measure

**Fig 2:**
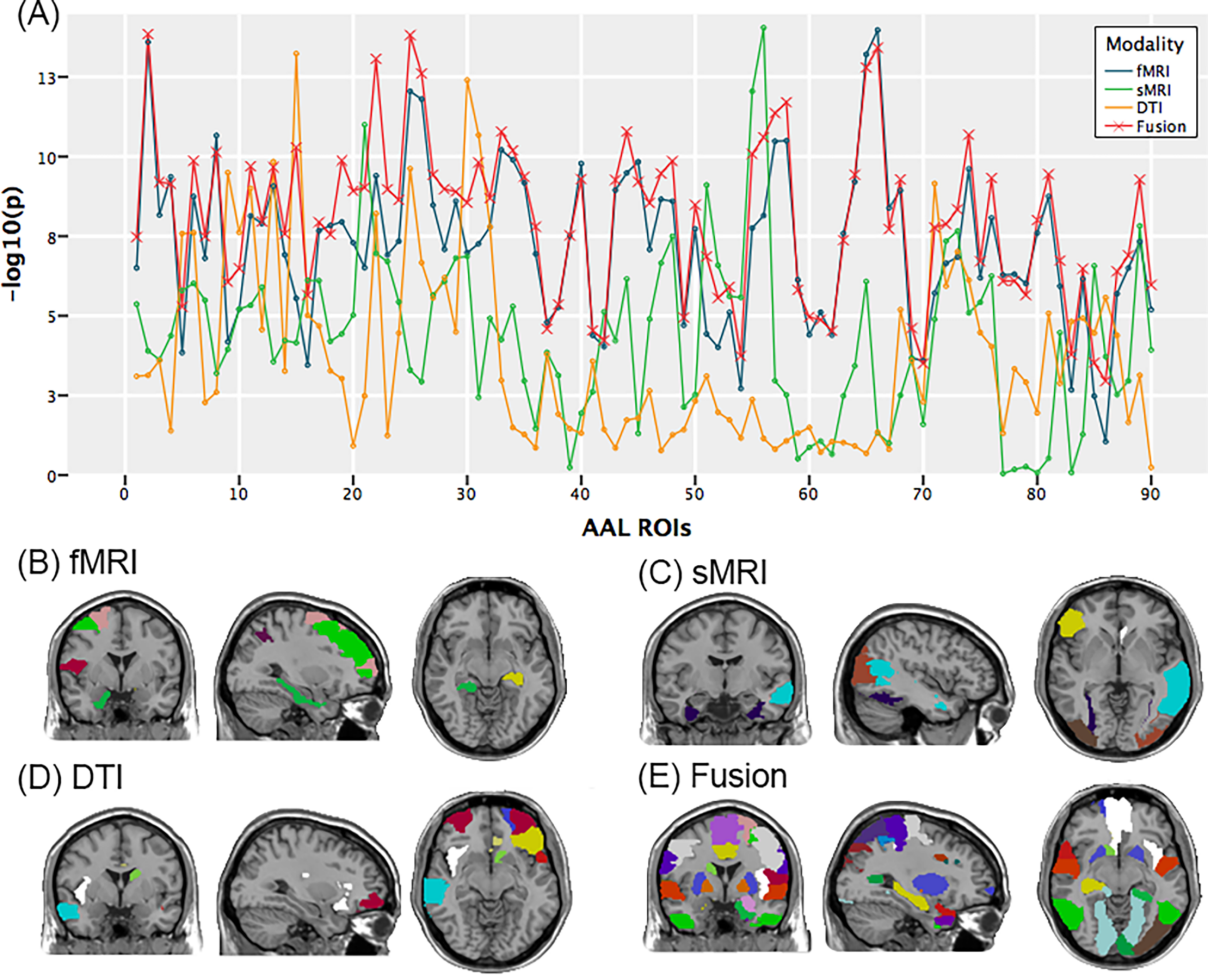
(A) Results of four kinds of measures with all components of fMRI. For visualization, the y label is -log_10_(*p*) rather than the *p* value. (B) 16 ROIs have the smallest p value for fMRI measure. (C) 9 ROIs have the smallest p value for sMRI measure. (D) 10 ROIs have the smallest p value for DTI measure. (E)55 ROIs have the smallest p value for fusion measure.

Firstly, we construct fusion features for each ROI which include 89 fMRI features, 2 sMRI features and 3 DTI features. Since there is a significant difference in duration of education between two groups, we perform the canonical correlation analysis between the fusion features and the duration of education for each ROI. That is, the maximum correlation between the linear combinations of fusion features and the duration of education. No ROI is found to have significant correlation under FDR correction. Thus, four types of measures, including three individual measures and one fusion measure, are constructed for each ROI. Next, we want to compare the performance of four types of measures. We calculate the *p* value of Wilk’s Lambda statistics from the fMRI measure (89 features), sMRI measure (2 features), DTI measure (3 features) and the fusion measure (94 features) individually. Fig 2(A) shows the results of all these four kinds of measures. For visualization, the y label is -log_10_(*p*) rather than the *p* value. Sixteen out of 90 ROIs have the smallest p value for fMRI measure; 9 out of 90 ROIs have the smallest p value for sMRI measure; 10 out of 90 ROIs have the smallest p value for DTI measure and 55 out of 90 ROIs have the smallest p value for fusion measure. The results are shown in Fig 2B–2E. It is easy to see that fusion measure outperforms other measures in that fusion measure shows more significant p-value for most ROIs.

As the dimension of fMRI is obviously higher than that of sMRI and DTI, it is possible that there is bias for the contribution of fMRI. In order to reduce the dimension of fMRI, we extract the principle component of fMRI features first for each ROI. The principal components serve as fMRI features then. S2(A) Fig is the mean contribution vs number of components. It is easy to see that the total contribution of the first 5 components is more than 50%. S2(B) Fig is the total contribution of the first 5 components for each ROI.

**Fig 3:**
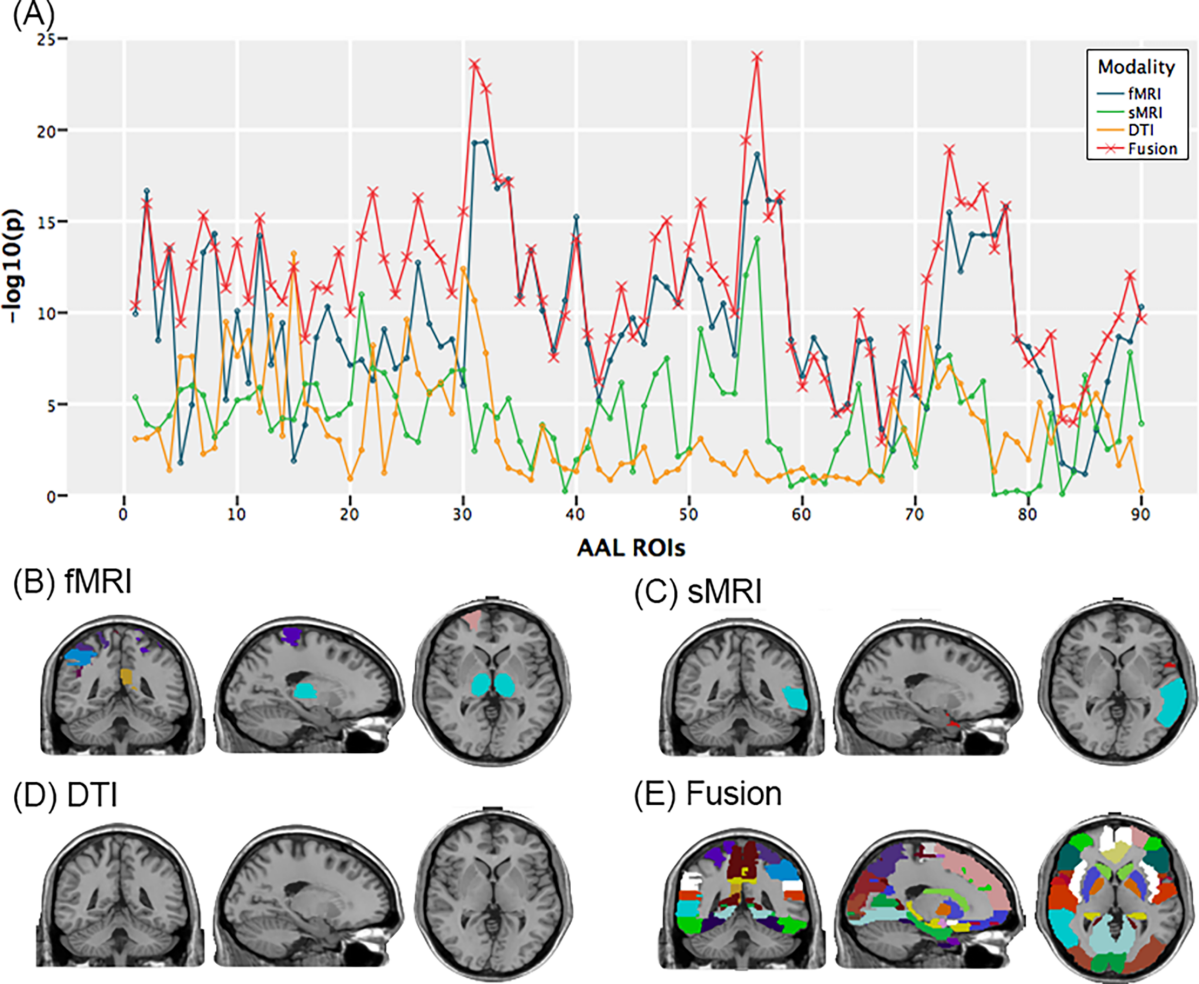
(A) Results of four kinds of measures with the 5 principle components of fMRI. For visualization, the y label is -log_10_(*p*) rather than the *p* value. (B) 10 ROIs have the smallest p value for fMRI measure. (C) 4 ROIs have the smallest p value for sMRI measure. (D) 0 ROIs have the smallest p value for DTI measure. (E) 76 ROIs have the smallest p value for fusion measure.

Next, we re-calculate the *p* value of Wilk’s Lambda statistics from the fMRI principle component measure (5 features), sMRI measure (2 features), DTI measure (3 features) and the fusion measure (10 features) for each ROI individually. Fig 3(A) shows the results of all these four kinds of measures. For visualization, the y label is -log_10_(*p*) rather than the *p* value. 10 out of 90 ROIs have the smallest p value for fMRI measure, 4 out of 90 ROIs have the smallest p value for sMRI measure, 0 out of 90 ROIs have the smallest p value for DTI measure, 76 out of 90 ROIs have the smallest p value for fusion measure. The results are shown in (Fig 3B–3E). It is also easy to see that fusion measure outperforms other measures in that fusion measure shows more significant p-value for most ROIs.

### Predictive power of different measures

In the present study, SVM is used to discriminate between subjects belonging to two different classes (i.e. patients and controls) for each of the 7 modal combinations. We hypothesize that the better the biomarker is, the higher the discrimination accuracy is.

Firstly, we compare the predictive power of four different measures with all components of fMRI. As we can see, the three-way fusion measurements achieve more accurate discrimination between schizophrenia patients and healthy controls. Specifically, for classifying schizophrenia from healthy controls, the fusion measure fMRI+sMRI+DTI achieve the highest classification accuracy of 86.52% among the 7 different modal combinations, and the best accuracy on measure of individual modality is 84.47% (when using fMRI).

**Fig 4:**
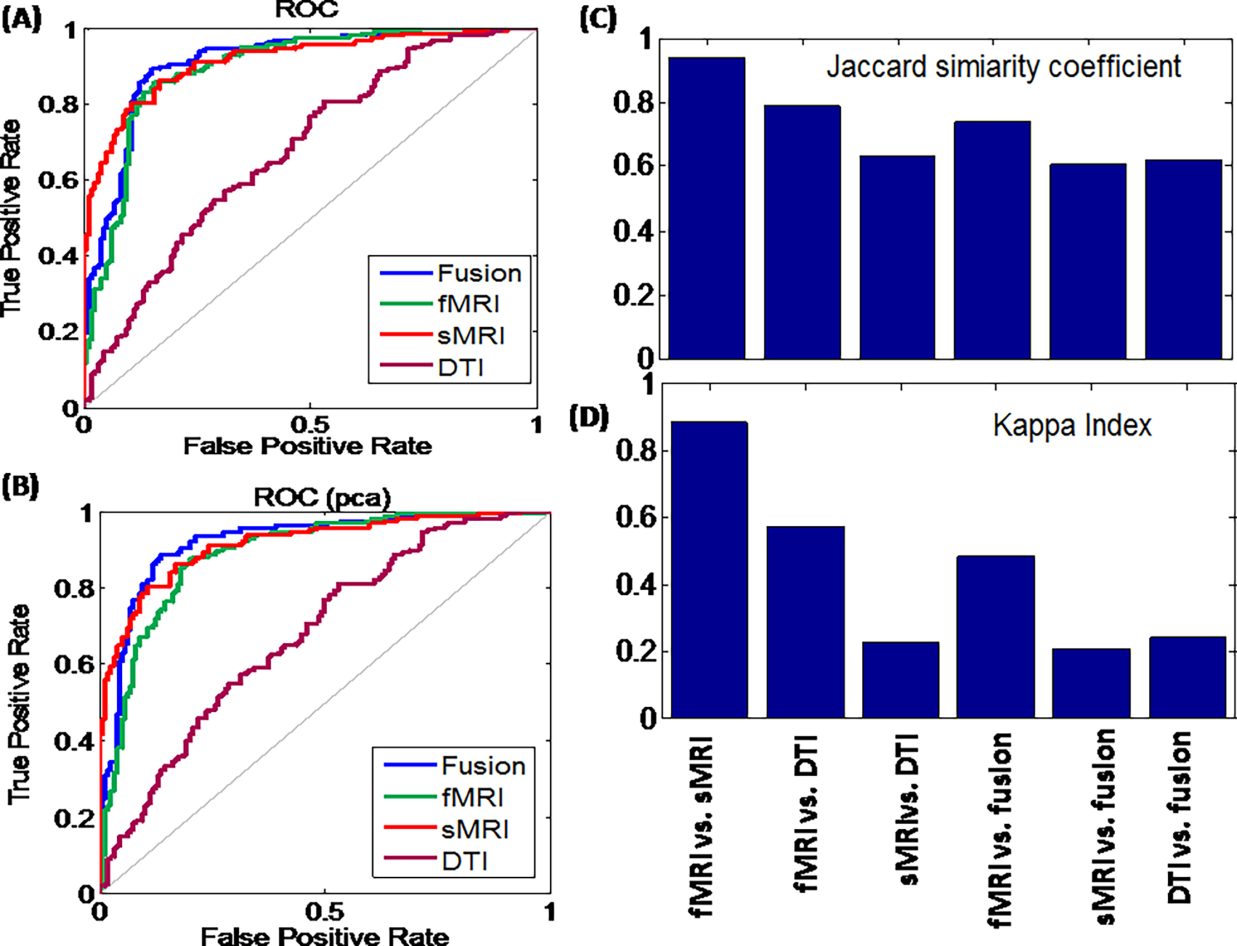
ROC curves of different modalities, for all components of fMRI (A) and for the first 5 components of fMRI (B). The Jaccard similarity coefficient (C) and Kappa index (D) for six different comparison.

Next, we compare the predictive power of different measures with the first 5 principle components of fMRI. It is also easy to see that the three way fusion measure (fMRI+sMRI+DTI) has the highest discriminative power (84.96%) than the measures in the other modal combinations. The statistical significance of these measures is determined by way of permutation testing (*n =* 100 permutations) and listed in Table 2. The ROC curves are shown in (Fig 4A and 4B). From the above results, it is easy to see that fusion of all three modalities provide the most plentiful information and the highest predictive accuracy of 86.52%. This work indicates that fusion of different modalities can improve the ability of distinguishing differences and provide optimized biomarkers in schizophrenia.

**Table 2:** Discrimination accuracy of four kinds of measures.

	Accuracy (mean/std)	p-value	Specificity	Sensitivity	AUC
fMRI+sMRI+DTI	a: 86.52% / 6%	<0.01	87.34%	85.98%	0.92
	b: 84.96% / 6.8%	<0.01	84.88%	85.26%	0.91
fMRI+sMRI	a: 85.86% / 6.4%	<0.01	85.81%	85.98%	0.92
	b: 84.61% / 6.7%	<0.01	84.26%	84.57%	0.91
fMRI+DTI	a: 83.94% / 6.8%	<0.01	84%	82.44%	0.9
	b: 81.57% / 6.7%	<0.01	81.43%	81.65%	0.89
fMRI	a: 84.47% / 6.9%	<0.01	84.6%	84.98%	0.91
	b: 81.43% / 6.7%	<0.01	81.83%	80.85%	0.89
sMRI+DTI	83.16% / 6.12%	<0.01	83.48%	82.27%	0.9
sMRI	83.94% / 6.21%	<0.01	83.7%	84.3%	0.9
DTI	65.76% / 8.1%	0.01	75%	56.52%	0.7

Note: a: all components of fMRI; b: first 5 principle components of fMRI; AUC: area under ROC curve.

Furthermore, we use statistical t-tests to compare these 10-fold cross-validation accuracies between individual modality and fusion measures with all components of fMRI. Significant differences are detected for three individual measures as compared with three-way fusion measures (p = 0.0261 for fMRI, p = 0.003 for sMRI, p<0.001 for DTI). Significant differences are also detected for two two-way fusion measures (p = 0.0049 for fMRI+DTI, p<0.001 for sMRI+DTI) while no difference is detected for fMRI+sMRI measures (p = 0.4527) as compared with three-way fusion measures. Similar results are obtained with the first 5 principle components of fMRI.

### Diversity of individual modalities in classification

Here, we quantitatively measure the discrimination similarity and diversity between any two different modalities, i.e., fMRI vs. sMRI, fMRIvs. DTI, sMRI vs. DTI. We also quantitatively measure the discrimination similarity and diversity between individual modalities vs fusion feature, i.e., fMRI vs. fusion, sMRI vs. fusion, and DTI vs. fusion by comparing their individual classification results. Both Jaccard similarity coefficient (J) and Kappa index (K) are used to measure the similarities and diversities, respectively. Small values on both indexes imply a low similarity and a high diversity on these two modalities. The results are shown in Fig 4C and 4D).

The Jaccard similarities (Kappa diversities) are 0.9422(0.8844), 0.7872(0.5744), 0.6322(0.2293) for fMRI vs. sMRI, fMRI vs. DTI, sMRI vs. DTI, respectively. On the other hand, the Jaccard similarities (Kappa diversities) are 0.7416(0.4833), 0.6049(0.2045), 0.6201(0.2385) for fMRI vs. fusion, sMRI vs. fusion, DTI vs. fusion, respectively. These results indicate that fMRI and sMRI have the highest similar information for classification among three modalities. They also indicate that the fMRI has the highest information for fusion feature.

### Contribution of different modalities

**Fig 5:**
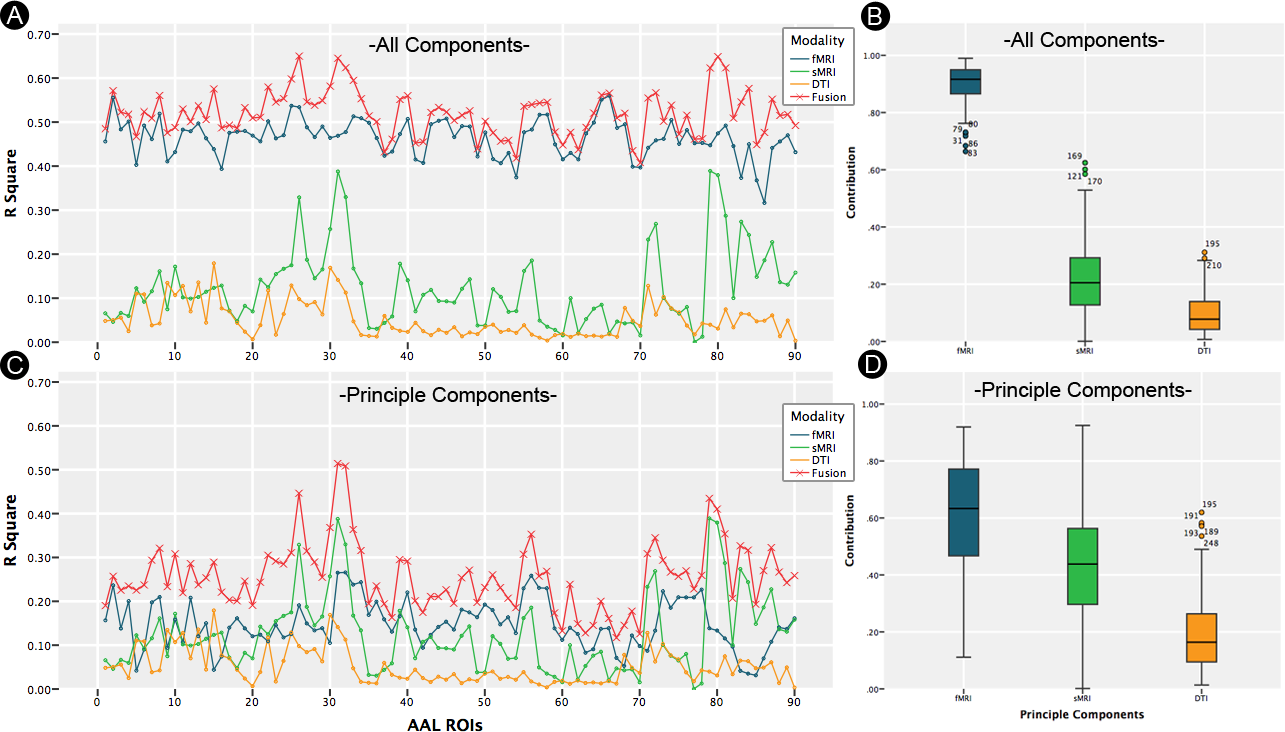
Goodness of fit for different measures with all components in fMRI (A) and the first 5 principle components in fMRI (C). The contribution of fMRI, sMRI, DTI to fusion data with all components in fMRI (B) and the first 5 components in fMRI (D).

We aim toquantitatively measure the contribution of each modality to fusion feature. Firstly, we use fMRI data with all components. There are 94 features in fusion data (89 fMRI features, 2 sMRI features, 3 DTI features). We calculate $R_{fusion}^{2}$, $R_{fMRI}^{2}$, $R_{sMRI}^{2}$, $R_{DTI}^{2}$ from four different models and calculate the contribution of each modality. Fig 5(A) is the goodness of fit for each model. Fig 5(B) shows the contributions of fMRI, sMRI and DTI to fusion data. It is easy to see that fMRI data is the most informative (90% contribution to the fusion feature) while DTI is the least informative (9.8% contribution to the fusion feature) among these three modalities.

Next, we extract the first 5 components of fMRI and there are 10 features in fusion data (5 fMRI features, 2 sMRI features, 3 DTI features). Fig 5(C) is the goodness of fit for each model. Fig 5(D) is the contribution of fMRI, sMRI and DTI to fusion data. It is easy to see that fMRI data is the most informative (59.8% contribution to the fusion feature) while DTI is the least informative (20% contribution to the fusion feature) among these three modalities as well.

## Discussion

Within this study, we developed a novel multivariate data fusion method to combine the resting state fMRI, sMRI and DTI without reducing the dimension or using the priors. By constructing the multi-index feature for each ROI, we calculated the p value of significant difference between groups for four different models (fMRI, sMRI, DTI, fusion). Our results showed that the three-way fusion feature has the smallest p value in most of the brain ROIs which indicates that we can increase the difference between the groups by integrating additional information. The results of discriminant analysis confirmed this conclusion which shows that each modality (fMRI, sMRI, and DTI) is indispensable for achieving good combination and classification. The fusion feature has the highest predictive accuracy of 86.12% while the best accuracy on measure of individual modality is only 84.47% when using fMRI. Our second job is to compare the similarity and diversity of individual modalities in classification. The fMRI and sMRI are very much similar both with Jaccard similarity coefficient (J = 0.9422) and with Kappa index (K = 0.8844). The fMRI has the highest information for fusion feature (J = 0.7416, K = 0.4833) among three individual modalities. At last, we find that fMRI feature has the greatest contribution of 90% to the fusion feature from the perspective of the goodness of fit. Our results show that the fusion feature can improve the ability to distinguish differences between groups, and fMRI is the most informative among these three individual modalities. We hope the method we proposed here will be potentially useful for identification of unique biomarkers of brain disorders.

A lot of studies have shown that biomarkers from different modalities may contain complementary information for the diagnosis of schizophrenia [47–51]. Several works have reported their works on combining different modalities of biomarkers [28, 40, 52, 53]. However, there have been only a few reports combining three or more types of brain imaging data to investigate brain disorders. For example, Correa examined changes that are related across fMRI, sMRI, and EEG data for SZ [54]. Groves compared Alzheimer’s patients and age-matched controls by combining gray matter (GM) density and three diffusion data measures (fractional anisotropy (FA), mean diffusivity (MD), and tensor mode) [25]. Other advances in multi-way data fusion include integrating multiple (task) fMRI data sets [55,56] from the same participant to specify common versus specific sources of activity to a greater degree than traditional general linear model-based approaches. However, there has been little report that combining resting state fMRI with other two or more different types of brain imaging data to study SZ.

Our proposed method has several advantages. First of all, our advantage is the multi-index feature we constructed. We work on the full-dimensional feature extracted from the large-scale original data, e.g., the whole brain FC constructed from the fMRI time series, whole brain GM and WM segmentation image from the sMRI scan and whole brain DTI measures such as FA, MD and RD. We utilize a full-dimensional feature-based approach providing a straightforward way to take advantage of data modeled without loss of information. Since the data between the different modalities is complementary, our results confirm that fusion feature of fMRI+sMRI+DTI has the most powerful discriminative accuracy.

Secondly, the multivariate statistical analysis we used make full use of the interactive information of the data without any modeling assumptions and priors. It is different from other hypotheses driven data fusion methods, such as multiple linear regression and confirmatory structural equation modeling, which are possible to miss important connectivity links that are not included in the a priori hypotheses and do not provide information about inter-relationships [57,58] It is also different from other data driven data fusion methods, such as jICA, mCCA, which are initialized from a PCA decomposition, some important information may be lost when performing PCA to do the dimension reduction[12, 16,23, 26].

We note that there is an alternative approach, called data integration, which distinguishes from “data fusion”. In most cases, data integration refers to analyzing each data type separately and then overlay them—thereby not allowing for any direct interaction between the data types. For example, there have been some attempts to utilize structural and functional information jointly (e.g., correlation of structural volumes with functional activation in certain regions) [59,60]. Voxelbased morphometric methods also provide a way to directly compare changes in relative gray matter amounts to changes in fMRI regions by overlapping the statistical maps created from each approach. Other existing approaches for data combination include constraining one modality with another, as EEG [61,62] or DTI [20, 63] being constrained by fMRI or sMRI data, or vice versa [64]. While these are powerful technique, a common limitation is that the potentially unrealistic assumptions which are fundamentally of a different nature than the known modality would be imposed upon the constrained data.

Thirdly, this is the first attempt to compare the similarity between these three modalities and the contribution of these modalities to the fusion feature. Our results showed that sMRI is similar with fMRI (J = 0.9422, K = 0.8844) while diverse with DTI (J = 0.6322, K = 0.2293). Our another promising result is that the fMRI data is the most informative (90% contribution to the fusion feature) while DTI is the least informative (9.8% contribution to the fusion feature) among these three modalities. Actually, it is easy to understand. fMRI measures the hemodynamic response related to neural activity in the brain dynamically which has better spatial resolution. It often scans for several minutes and contains more information compared with sMRI. As for DTI, we just integrate three feathers FA, RD, MD to the fusion features, it is certainly less informative than fMRI feature. Of course, we can fully integrate the DTI data into our approach, as we do for fMRI. Due to various constrains on DTI such as the quality issue for fibers with small fiber numbers, we here only include FA, RD and MD in our analysis.

Schizophrenia is a complex disease and considered to be caused by the interplay of a number of genetic and environmental factors. Besides these neuroimaging techniques, there are also some biological or genetic biomarkers developed for diagnosis of schizophrenia. Even though there are around 30 schizophrenia-associated loci been identified through GWAS [65–68], they cannot be served as diagnosis markers. Fusion of imaging findings and genetic information may provide an alternative way for biomarker discovery, which cannot only contribute to a better understanding of biological mechanisms on brain structure and function but also have the potential to improve the diagnosis and treatments of complex diseases [69, 70]. By comparing the contribution of each imaging modality to the fusion data and investigate these imaging modalities, this study may provide important experience and technology for further studies.

The study’s primary limitation is that at the time of scanning, all of the schizophrenia patients are medicated, and correspondingly, symptomatically stable. Although we find no evidence for a significant impact of medication, it is possible that anti-psychotic exposure, or even other variables not considered here, may still have contributed to the classification in an as yet undetectable way, potentially confounding the inference one can draw from the successful discriminator. Another limitation, applicable to any study with access restricted to their own sample, is that as a single-centre, cross-sectional, study we are unable to make inference regarding the generalizability across different research centres for the validity of our method. The third limitation, fusion of multi-modality can improve the ability of distinguishing differences between groups and might be assisting in further diagnosis of schizophrenia. However, we cannot provide more evidence about this additional cross-information. We hope that we will know more about it with other new method.

## Supporting information

S1 File Supplemental Materials. (DOCX)

S1 Fig The flowchart of the pattern recognition. (TIF)

S2 Fig (A) The mean contribution vs number of components. It is easy to see that the total contribution of the first 5 components is more than 50%. (B) The total contribution of the first 5 components for each ROI.
(TIF)
