# Supplementary material for: Combining multi-modality data for searching biomarkers in schizophrenia
Source: PLoS One. 2018 Feb 1;13(2):e0191202. doi: 10.1371/journal.pone.0191202 (PMC5794071; doi:10.1371/journal.pone.0191202)
Supplement: S1 File — (DOCX) [file pone.0191202.s001.docx]

Supplemental Materials

**Definition of** $\lambda_{1}$，$\lambda_{2}$, $\lambda_{3}$

In fibrous tissues including white matter, water diffusion is relatively unimpeded in the direction parallel to the fiber orientation. Conversely, water diffusion is highly restricted and hindered in the directions perpendicular to the fibers. Thus, the diffusion in fibrous tissues is anisotropic. Once we have measured the voxel of restricted tissue from six or more directions, we can use the information from our calculated diffusion tensor to describe what is happening in the water restricted voxel (ellipsoid model). These diffusion tensors could be used to describe the diffusion of water molecules with a Gaussian model. Technically, it is proportional to the covariance matrix of a three-dimensional Gaussian distribution that models the displacements of the molecules by the diffusion time. The diffusion tensors is a 3×3 covariance matrix,

$D=\left[\begin{matrix} D_{xx} D_{xy} D_{xz} \\ D_{yx} D_{yy} D_{yz} \\ D_{zx} D_{zy} D_{zz} \end{matrix}\right]$

these matrix properties mean that it has 3 orthogonal (mutually perpendicular) eigenvectors and three positive eigenvalues. The major eigenvector of the diffusion tensor points in the principal diffusion direction (the direction of the fastest diffusion). In anisotropic fibrous tissues the major eigenvector also defines the fiber tract axis of the tissue, and thus the three orthogonal eigenvectors can be thought of as a local fiber coordinate system. The three positive eigenvalues of the tensor: $\lambda_{1}$，$\lambda_{2}$ and  $\lambda_{3}$, give the diffusivity in the direction of each eigenvector. The eigenvalue magnitudes may be affected by changes in local tissue microstructure with many types of tissue injury, disease or normal physiological changes (i.e., aging). Together, the eigenvectors and eigenvalues define an ellipsoid that represents an isosurface of (Gaussian) diffusion probability. Thus, the diffusion tensor is a sensitive probe for characterizing both normal and abnormal tissue microstructure.

**Table S1: The names and abbreviations of the regions of interest (ROIs)**

| Regions Abbr. | | Regions | Abbr. |
| --- | --- | --- | --- |
| Amygdala  Angular gyrus  Anterior cingulate gyrus  Calcarine cortex  Caudate  Cuneus  Fusiform gyrus  Heschl gyrus  Hippocampus  Inferior occipital gyrus  Inferior frontal gyrus (opercula)  Inferior frontal gyrus(triangular)  Inferior parietal lobule  Inferior temporal gyrus  Insula  Lingual gyrus  Middle cingulate gyrus  Middle occipital gyrus  Middle frontal gyrus  Middle temporal gyrus  Olfactory  Orbitofrontal cortex (inferior)  Orbitofrontal cortex (medial) | AMYG  ANG  ACG  CAL  CAU  CUN  FFG  HES  HIP  IOG  IFGoperc  IFGtriang  IPL  ITG  INS  LING  MCG  MOG  MFG  MTG  OLF  ORBinf  ORBmed | Orbitofrontal cortex (middle)  Orbitofrontal cortex (superior)  Pallidum  Paracentral lobule  Parahippocampal gyrus  Postcentral gyrus  Posterior cingulate gyrus  Precentral gyrus  Precuneus  Putamen  Rectus gyrus  Rolandic operculum  Superior occipital gyrus  Superior frontal gyrus (dorsal)  Superior frontal gyrus (medial)  Superior parietal gyrus  Superior temporal gyrus  Supplementary motor area  Supramarginal gyrus  Temporal pole (middle)  Temporal pole (superior)  Thalamus | ORBmid  ORBsup  PAL  PCL  PHG  PoCG  PCG  PreCG  PCUN  PUT  REC  ROL  SOG  SFGdor  SFGmed  SPG  STG  SMA  SMG  TPOmid  TPOsup  THA |
