# Supplementary figures and images for: Combining multi-modality data for searching biomarkers in schizophrenia
Source: PLoS One. 2018 Feb 1;13(2):e0191202. doi: 10.1371/journal.pone.0191202 (PMC5794071; doi:10.1371/journal.pone.0191202)

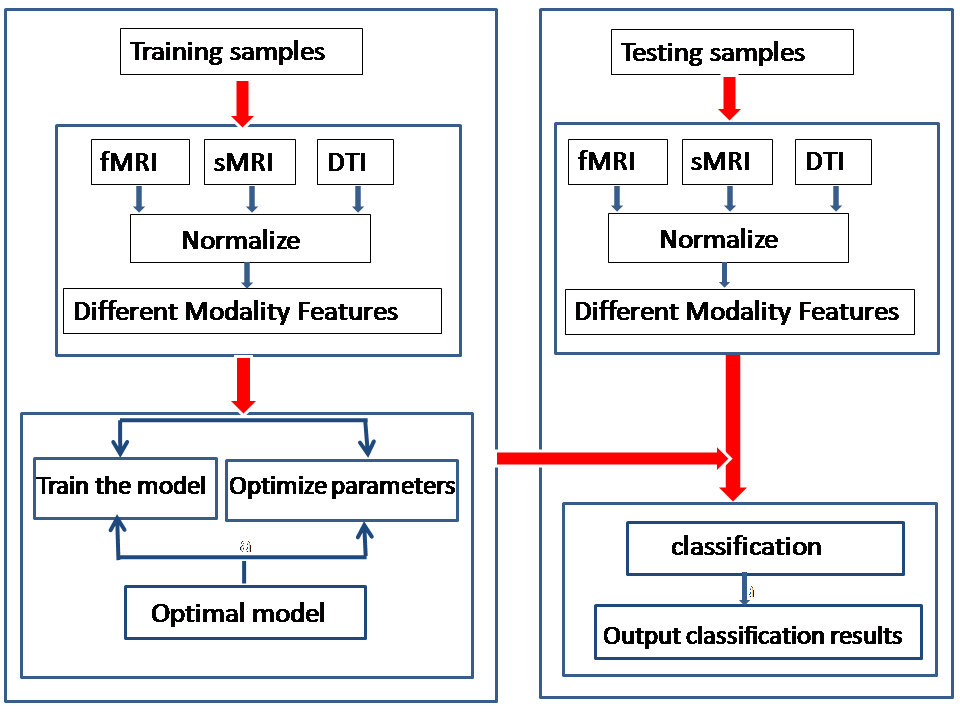

Supplement: S1 Fig — (TIF) [file pone.0191202.s002.tif]

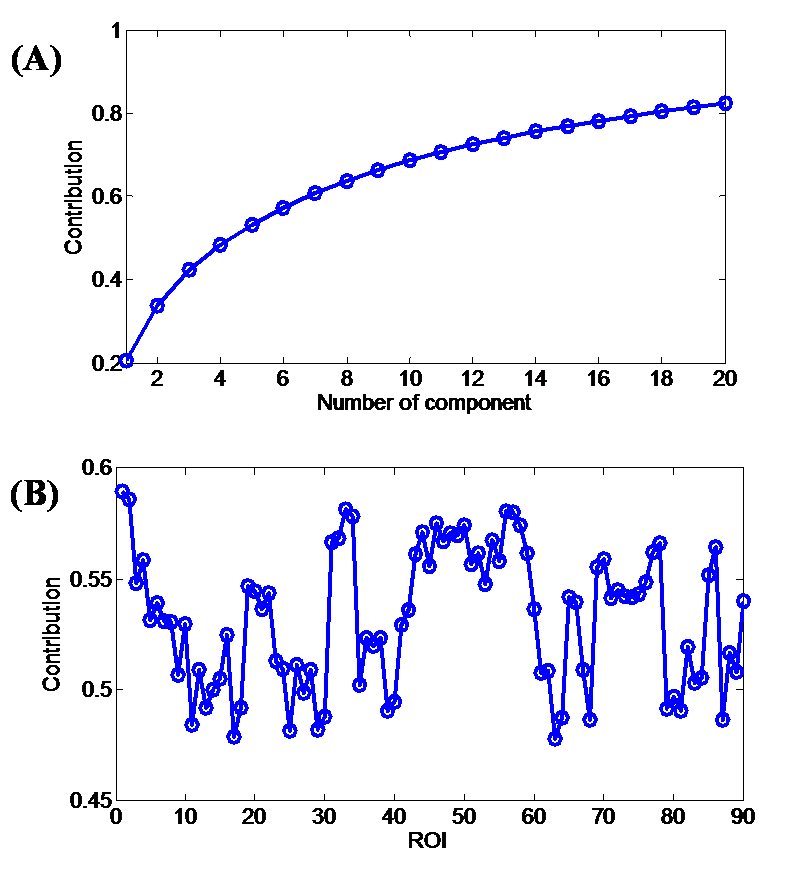

Supplement: S2 Fig — (A) The mean contribution vs number of components. It is easy to see that the total contribution of the first 5 components is more than 50%. (B) The total contribution of the first 5 components for each ROI. (TIF) [file pone.0191202.s003.tif]
